# Biomarker-defined pathways for incident type 2 diabetes and coronary heart disease—a comparison in the MONICA/KORA study

**DOI:** 10.1186/s12933-020-01003-w

**Published:** 2020-03-12

**Authors:** Cornelia Huth, Alina Bauer, Astrid Zierer, Julie Sudduth-Klinger, Christa Meisinger, Michael Roden, Annette Peters, Wolfgang Koenig, Christian Herder, Barbara Thorand

**Affiliations:** 1 Institute of Epidemiology, Helmholtz Zentrum München–German Research Center for Environmental Health (GmbH), Ingolstädter Landstraße 1, 85764 Neuherberg, Germany; 2 German Center for Diabetes Research (DZD), München-Neuherberg, Germany; 3 Tethys Bioscience Inc., Emeryville, CA USA; 4 Chair of Epidemiology, Ludwig-Maximilians-Universität München, UNIKA-T Augsburg, Augsburg, Germany; 5 Independent Research Group Clinical Epidemiology, Helmholtz Zentrum München–German Research Center for Environmental Health (GmbH), Neuherberg, Germany; 6 Institute for Clinical Diabetology, German Diabetes Center, Leibniz Center for Diabetes Research at Heinrich Heine University Düsseldorf, Düsseldorf, Germany; 7 Division of Endocrinology and Diabetology, Medical Faculty, Heinrich Heine University Düsseldorf, Düsseldorf, Germany; 8 German Centre for Cardiovascular Research (DZHK), Partner Site Munich Heart Alliance, Munich, Germany; 9 Institute of Epidemiology and Medical Biometry, University of Ulm, Ulm, Germany; 10 Deutsches Herzzentrum München, Technische Universität München, Munich, Germany

**Keywords:** Type 2 diabetes, Coronary heart disease, Case-cohort study, Pathways, Biomarker, Fetuin-A, IGFBP-2, Lp(a), NT-proBNP, Troponin I

## Abstract

**Background:**

Biomarkers may contribute to our understanding of the pathophysiology of various diseases. Type 2 diabetes (T2D) and coronary heart disease (CHD) share many clinical and lifestyle risk factors and several biomarkers are associated with both diseases. The current analysis aims to assess the relevance of biomarkers combined to pathway groups for the development of T2D and CHD in the same cohort.

**Methods:**

Forty-seven serum biomarkers were measured in the MONICA/KORA case-cohort study using clinical chemistry assays and ultrasensitive molecular counting technology. The T2D (CHD) analyses included 689 (568) incident cases and 1850 (2004) non-cases from three population-based surveys. At baseline, the study participants were 35–74 years old. The median follow-up was 14 years. We computed Cox regression models for each biomarker, adjusted for age, sex, and survey. Additionally, we assigned the biomarkers to 19 etiological pathways based on information from literature. One age-, sex-, and survey-controlled average variable was built for each pathway. We used the R^2^_PM_ coefficient of determination to assess the explained disease risk.

**Results:**

The associations of many biomarkers, such as several cytokines or the iron marker soluble transferrin receptor (sTfR), were similar in strength for T2D and CHD, but we also observed important differences. Lipoprotein (a) (Lp(a)) and N-terminal pro B-type natriuretic peptide (NT-proBNP) even demonstrated opposite effect directions. All pathway variables together explained 49% of the T2D risk and 21% of the CHD risk. The insulin-like growth factor binding protein 2 (IGFBP-2, IGF/IGFBP system pathway) best explained the T2D risk (about 9% explained risk, independent of all other pathway variables). For CHD, the myocardial-injury- and lipid-related-pathways were most important and both explained about 4% of the CHD risk.

**Conclusions:**

The biomarker-derived pathway variables explained a higher proportion of the T2D risk compared to CHD. The ranking of the pathways differed between the two diseases, with the IGF/IGFBP-system-pathway being most strongly associated with T2D and the myocardial-injury- and lipid-related-pathways with CHD. Our results help to better understand the pathophysiology of the two diseases, with the ultimate goal of pointing out targets for lifestyle intervention and drug development to ideally prevent both T2D and CHD development.

## Background

Type 2 diabetes (T2D) and coronary heart disease (CHD) are among the most common chronic diseases in Western industrialized countries and cardiovascular diseases (CVD) are the main cause of death [1]. Although T2D is usually not a direct cause of death, patients with diabetes have a significantly increased risk of CVD and microvascular complications such as kidney disease [2, 3]. Among the most important risk factors for both diseases are an unhealthy lifestyle (e.g. too little exercise, smoking, and unhealthy diet) and disorders such as obesity, high blood pressure, dyslipidemia, subclinical inflammatory processes, and insulin resistance [4–7]. Michael P. Stern suggested in 1995 that T2D and CVD have common genetic and environmental antecedents, coining the term of the ‘common soil’ from which both conditions arise [8]. The extent and components of the common roots are still a field of active research, especially since it was discovered that there are also factors that protect against T2D, but increase the risk of myocardial infarction (MI) [9].

Our study examines the common soil hypothesis by investigation of biomarkers, which reflect diverse etiologic pathways. Serum and plasma biomarkers are increasingly used to elucidate pathophysiological changes leading to T2D and CHD. Biomarkers are objectively measurable and are not subject to recall bias. Numerous examples show the benefit of biomarkers for etiologic research [10, 11]. However, most published studies have investigated either T2D [12] or CHD [13], so that the results of the two diseases cannot be directly compared.

The current investigation was embedded within the Monitoring of Trends and Determinants in Cardiovascular Disease (MONICA) Augsburg study, which was initiated in the early 1980s by the World Health Organization to study risk factors of premature CVD in regionally defined communities by a standardized protocol. In previous analyses, several biomarkers have already been investigated with respect to their association with either T2D (e.g. [14–16]) or CHD (e.g. [17–20]) or both [6]. Unlike the investigation of single candidate biomarkers, the simultaneous analysis of multiple biomarkers reflecting different pathomechanisms allows to compare the association of different metabolic pathways with disease development. This information may be valuable in order to target the most relevant pathways with drug treatments or lifestyle interventions. In contrast to previous analyses within the same cohort, this study uses a multi-marker approach including integration of biomarkers into pathway variables, to directly compare associations with both outcomes for a uniformly restricted 14-year follow-up period. Thus, we aim to assess and compare the relevance of 47 single biomarkers and 19 etiologic pathways derived from these biomarkers for the development of T2D and CHD simultaneously in the same cohort.

## Methods

### Study population

The design and all procedures of this prospective case-cohort study within the population-based MONICA/KORA Augsburg cohort have been described in detail before [21]. Briefly, three independent cross-sectional population-based surveys were performed within the MONICA Augsburg project in 1984/85 (survey S1), 1989/90 (S2) and 1994/95 (S3) in Augsburg and two adjacent counties (Germany). The total number of participants was 13,427 (6725 men, 6702 women) aged 25–64 (S1) or 25–74 years (S2, S3). Information on sociodemographic and lifestyle variables at baseline was collected through standardized interviews. In addition, standardized medical examinations were performed. All participants were prospectively followed within the framework of the Cooperative Health Research in the Region of Augsburg (KORA). Due to the low incidence of T2D and CHD under the age of 35, we restricted the source population to the 10,718 persons (5382 men and 5336 women) between 35 and 74 years of age at baseline. Details on the selection of study participants are shown in Additional file 1: Figure S1. The follow-up period was restricted to a maximum of 14 years because the maximum follow-up for the S3 participants was 14–15 years.

The incidence of T2D was assessed using a written follow-up questionnaire sent to all participants of the three baseline surveys in 1997/98, 2002/03, and 2008/09. Furthermore, all S1 participants were invited to a follow-up examination in 1987/88. Self-reported incident T2D status and the date of diagnosis were validated by a questionnaire mailed to the treating physician or medical chart review. Only participants for whom the treating physician clearly reported a diagnosis of T2D or for whom a diagnosis of T2D was mentioned in the medical records or who were taking antidiabetic medication were classified as cases. The mean follow-up time ± standard deviation (SD) was 12.0 ± 3.5 years, the median was 14.0 years. The T2D case-cohort study included only participants without prevalent diabetes at baseline. It comprised a randomly drawn subcohort of 1991 individuals (of whom *n* = 141 developed incident T2D) plus all 548 additional incident T2D cases.

CHD was defined as non-fatal MI as well as coronary death and sudden death (International Classification of Disease 9^th^ Revision (ICD 9): 410–414 and 798). Until December 2000, the diagnosis of a major, non-fatal MI and coronary death was based on the MONICA algorithm [22], where a diagnosis of a major CHD event was based on symptoms, cardiac enzymes (creatine kinase, aspartate aminotransferase, and lactate dehydrogenase), and serial changes from 12-lead ECGs evaluated by Minnesota coding [23], necropsy results, and history of CHD in fatal cases. Since January 1, 2001 all patients with MI diagnosed according to ESC (European Society of Cardiology) and ACC (American College of Cardiology) criteria were included [24]. Incident events were identified through the follow-up questionnaires or through the MONICA/KORA myocardial infarction registry [25]. The registry covers all acute MI cases occurring in the study area in patients up to 74 years. Questionnaire-assessed self-reported CHD events which were not covered by the MONICA/KORA myocardial infarction registry were validated by information from hospital discharge letters or from the treating physician. Coronary deaths were validated by autopsy reports, death certificates, chart reviews, and information from the last treating physician. The mean follow-up time ± SD was 12.6 ± 3.0 years, the median was 14.0 years. The CHD case-cohort study included only participants without a history of MI at baseline. It comprised a randomly drawn subcohort of 2163 individuals (of whom *n* = 159 developed incident CHD) plus all 409 additional incident CHD cases.

### Biomarker measurements, imputation, and definition of pathway variables

During the baseline examinations, a non-fasting venous blood sample was collected while sitting. Samples were centrifuged within 120 min, refrigerated at 4 to 8 °C and shipped on refrigerant packaging within 2–4 h to the laboratory of the Augsburg Central Hospital (now university hospital of Augsburg) for measurement of serum high density lipoprotein (HDL) cholesterol, total cholesterol, and uric acid. All other 44 biomarkers were measured from serum samples stored at − 80 °C.

On average, 16% of the biomarker data were missing, with a large variability between the biomarkers (minimum: creatinine, 0%; maximum: 25-hydroxycholecalciferol (25(OH)D), 30%). In order to enable unbiased analyses and to use the available data most efficiently, we replaced missing biomarker values in each of the two case-cohort datasets, using 20-fold multiple imputation by chained equations (MICE) (R version 3.2.3 and R package MICE version 2.25) [26–29].

Biomarker measurements with a right-skewed distribution were ln-transformed and all biomarkers were (0,1)-standardized. The measurement methods, coefficients of variation, proportions of missing values pre-imputation and decisions on ln-transformations are summarized in Additional file 1: Table S1 for all analyzed 47 biomarkers.

**Fig. 1:**
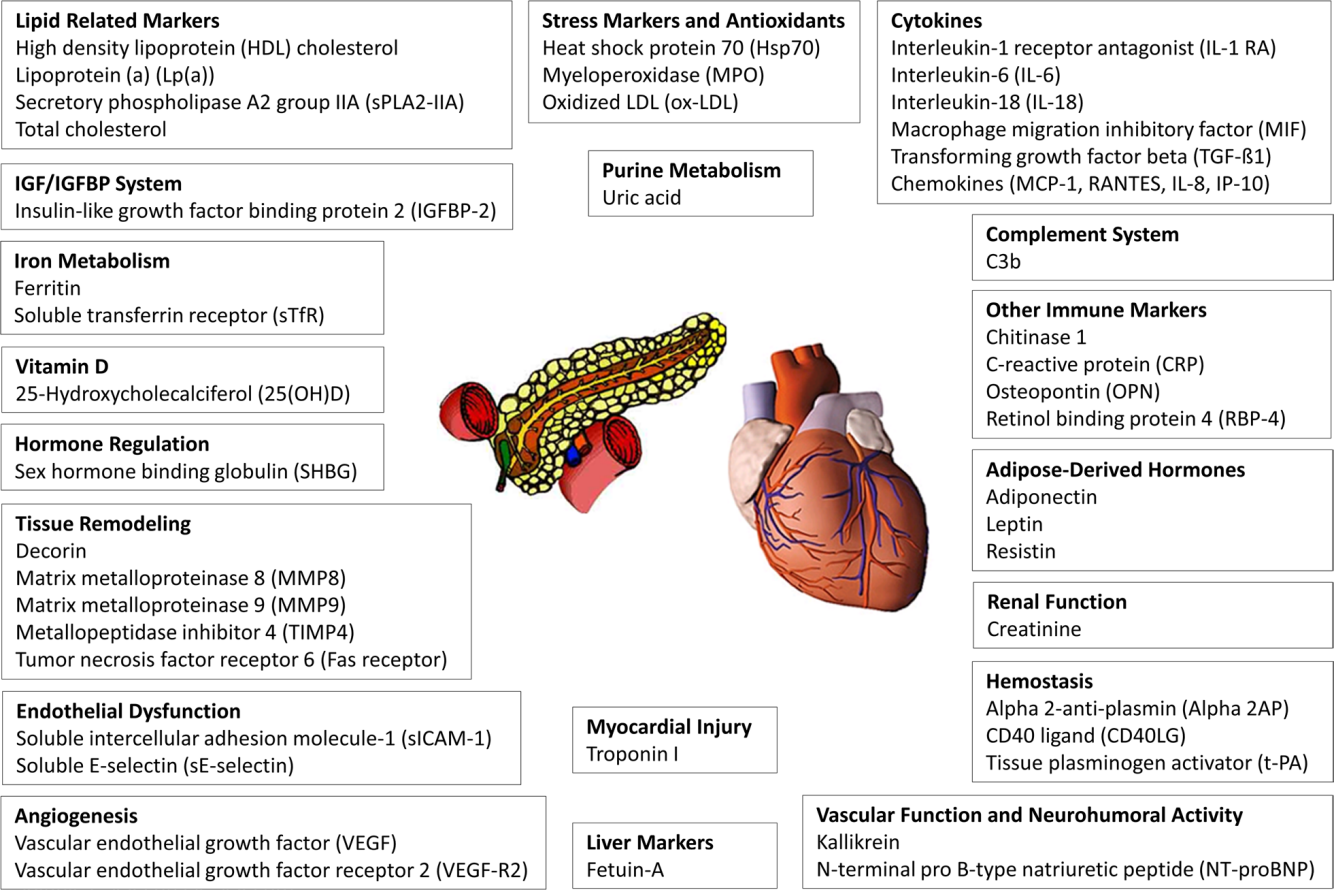
Overview of investigated biomarkers that were grouped in pathway variables according to pathophysiological processes

The biomarkers were selected with regard to their potential importance for either T2D or CHD pathophysiology based on prior knowledge from experimental and epidemiological studies. The current study used all available biomarker data from the two case-cohort studies for simultaneous analysis of both diseases in the form of a secondary data analysis. For this purpose, the biomarkers were grouped according to pathophysiological aspects; these biomarker groups are called ‘pathway variables’ in the following. Each biomarker was assigned to one pathway based on information from literature (Fig. 1). This approach leads to pathway groups differing in the number of included biomarkers. Therefore, one age, sex, and survey controlled ‘average’ variable was built for each of the 19 considered pathways in each of the 20 imputed datasets as follows: First, 47 linear regression models, each containing one standardized biomarker variable as dependent variable and age, sex, and survey as explanatory variables, were computed separately in the subcohort of the T2D and CHD case-cohort studies. Based on these results, biomarker residuals free of variability caused by age, sex, and survey were calculated in the complete case-cohort studies using the beta-estimates from the subcohort regression analyses. Third, the residuals of inversely associated biomarkers were multiplied by (− 1), allowing different decisions for T2D and CHD; the association directions were determined based on hazard ratios (HR) adjusted for age, sex, and survey. Fourth, the biomarker residual variables per pathway were summed and (0,1)-standardized again in order to yield the final pathway variables.

### Statistical analysis

Descriptive analyses of baseline characteristics were computed for cases and non-cases using the SAS procedure SURVEYMEANS (SAS Version 9.3 Institute Inc., Cary, NC, USA). To account for the case-cohort design, weighting was performed using the survey- and sex-specific sampling weights. Medians and interquartile ranges were calculated as median of percentiles over multiple results of 20 imputations; proportions were calculated as arithmetic means over the 20 imputation results.

Correlations between the age, sex, and survey adjusted biomarker residuals and between the pathway variables were investigated separately in the subcohorts of the T2D and CHD case-cohort studies by Pearson’s coefficients. Fisher’s Z-transformation and re-transformation was used to compute average correlation coefficients over the 20 imputed datasets [30, 31].

To assess the relation of the (0,1)-standardized biomarker variables with incident T2D and incident CHD, Cox proportional hazards regression models adjusted for age, sex, and survey were built. Regression coefficients were calculated using Barlow’s weighting method to account for the case-cohort design [32]. Robust variance estimation was performed to obtain standard error estimates for the parameter estimates [33]; incorporation of the additional variation due to imputation was achieved by using Rubin’s rules for multiple imputation [34].

For calculation of the explained time-to-event outcome variability (for simplicity called risk in the following), the coefficient of determination ${\text{R}}^{ 2}_{\text{PM}}$ according to Kent and O’Quigley was used, which takes the time-to-event data structure of Cox models into account [35, 36]. In general, the coefficient of determination R^2^ determines the ratio of the outcome variability explained by the model to the total variability. The variability explained by the model was estimated separately in each imputed dataset and combined by calculating the arithmetic mean. In order to determine the T2D and CHD outcome variabilities explained by all pathway variables, ${\text{R}}^{ 2}_{\text{PM}}$ was calculated for the full Cox models containing all 19 pathway variables. To assess the explained variability of each individual pathway, two approaches were chosen. Firstly, ${\text{R}}^{ 2}_{\text{PM}}$ of all Cox models containing exactly one of the 19 pathway variables was computed (called univariate assessment in the following). Secondly, the absolute ${\text{R}}^{ 2}_{\text{PM}}$ difference between full models and models in which one pathway variable was excluded was computed in order to assess the contribution of single pathway variables on top of the other 18 pathway variables (called independent assessment in the following). More details on the rationale of our analytical approach are given in Additional file 1: Text S1. All statistical analyses were performed with R version 3.6.1 unless specified otherwise. Test results with two-sided *p* value < 0.05 were considered statistically significant.

## Results

### Baseline characteristics

Table 1 shows the characteristics of the study participants at the time of the baseline surveys. For both outcomes, the cases comprised more men than women, they were more likely to be physically inactive, to suffer from actual hypertension and to be current or former smokers. Moreover, the cases were on average older, especially the incident CHD cases, and they had a higher BMI, which was more pronounced for the T2D cases.

**Table 1:** Baseline characteristics of cases and non-cases in the T2D and CHD case-cohort studies

Characteristics at baseline	Incident T2D		Incident CHD	
	Cases n = 689	Non-cases n = 1850	Cases n = 568	Non-cases n = 2004
Male (%)	57.0	48.2	73.1	46.9
Age (years)	57.0 (50.0; 64.0)	51.0 (43.0; 60.0)	62.0 (55.0; 68.0)	52.0 (43.0; 60.0)
Survey 1 (%)	23.8	30.2	24.6	28.8
Survey 2 (%)	40.3	36.0	42.8	36.3
Survey 3 (%)	35.8	33.8	32.6	35.0
BMI (kg/m^2^)	29.5 (27.2; 32.7)	26.3 (24.0; 29.1)	27.9 (25.8; 30.6)	26.5 (24.2; 29.5)
Physically inactive (%)	70.2	60.0	70.6	61.7
Actual hypertension^a^ (%)	66.5	39.9	68.0	40.7
Current smoking (%)	26.3	24.1	32.4	24.2
Former smoking (%)	31.7	27.8	35.6	27.3
Never smoking (%)	42.1	48.0	32.0	48.5

Categorical variables are presented as proportions and continuous variables as medians (25th percentile; 75th percentile)

^a^ Hypertension (ISH-WHO 1999) or medically treated with known hypertension

The unadjusted baseline values of all 47 biomarkers are shown in Additional file 1: Table S2 separately for incident T2D cases and non-cases as well as incident CHD cases and non-cases.

### Associations of single biomarkers with incident T2D and incident CHD

**Fig. 2:**
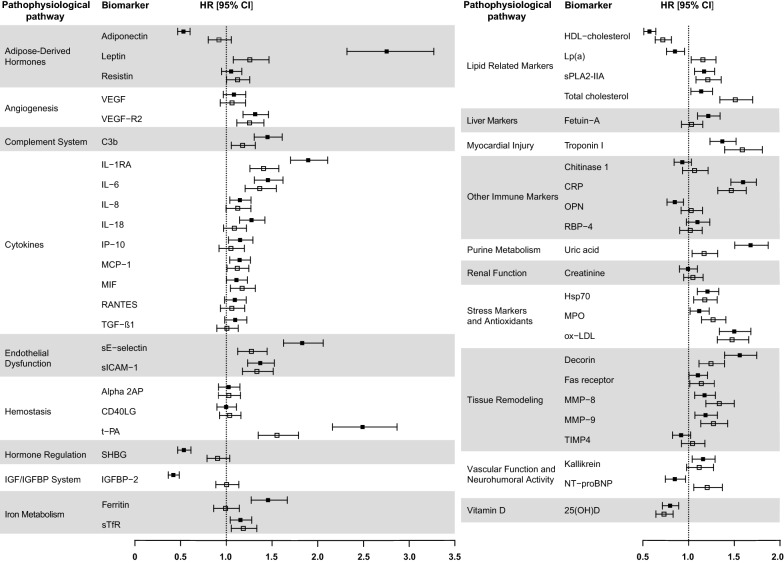
Age, sex, and survey adjusted hazard ratios (HR) with 95% confidence intervals (95% CI) per standard deviation (SD) increase in biomarker concentration for incident T2D (black boxes) and incident CHD (white boxes), sorted alphabetically by pathway group

The age, sex, and survey adjusted associations of the single biomarkers with incident T2D and incident CHD, sorted according to the assigned pathways, are shown in Fig. 2 and Additional file 1: Table S3. Overall, more biomarkers were significantly associated with T2D (n = 37) than with CHD (n = 28) and the T2D associations were on average stronger. Except for resistin, all biomarkers associated with CHD were also associated with T2D. The direction of the association was consistent for 25 of the 27 biomarkers that were associated with both diseases, but differed for lipoprotein (a) (Lp(a)) and N-terminal pro B-type natriuretic peptide (NT-proBNP). These two biomarkers were inversely associated with T2D and positively with CHD. For T2D, the adipose-derived hormone leptin (HR per SD = 2.75 [95% CI 2.31; 3.26]), the hemostasis marker tissue plasminogen activator (t-PA; 2.48 [2.16; 2.86]), and the insulin-like growth factor (IGF) binding protein 2 (IGFBP-2) (0.42 [0.37; 0.49]) demonstrated the strongest associations. For CHD, the strongest associations were observed for the myocardial injury marker troponin I (1.59 [1.39; 1.81]), t-PA (1.55 [1.35; 1.79]) and the lipid related marker total cholesterol (1.51 [1.34; 1.70]).

Pearson’s correlations were lower than 0.8 between the single age, sex, and survey controlled biomarker variables (Additional file 2: Figure S4) and were lower than 0.6 between the grouped pathway variables (Additional file 1: Figures S2 and S3).

### T2D and CHD risk explained by pathway variables

**Fig. 3:**
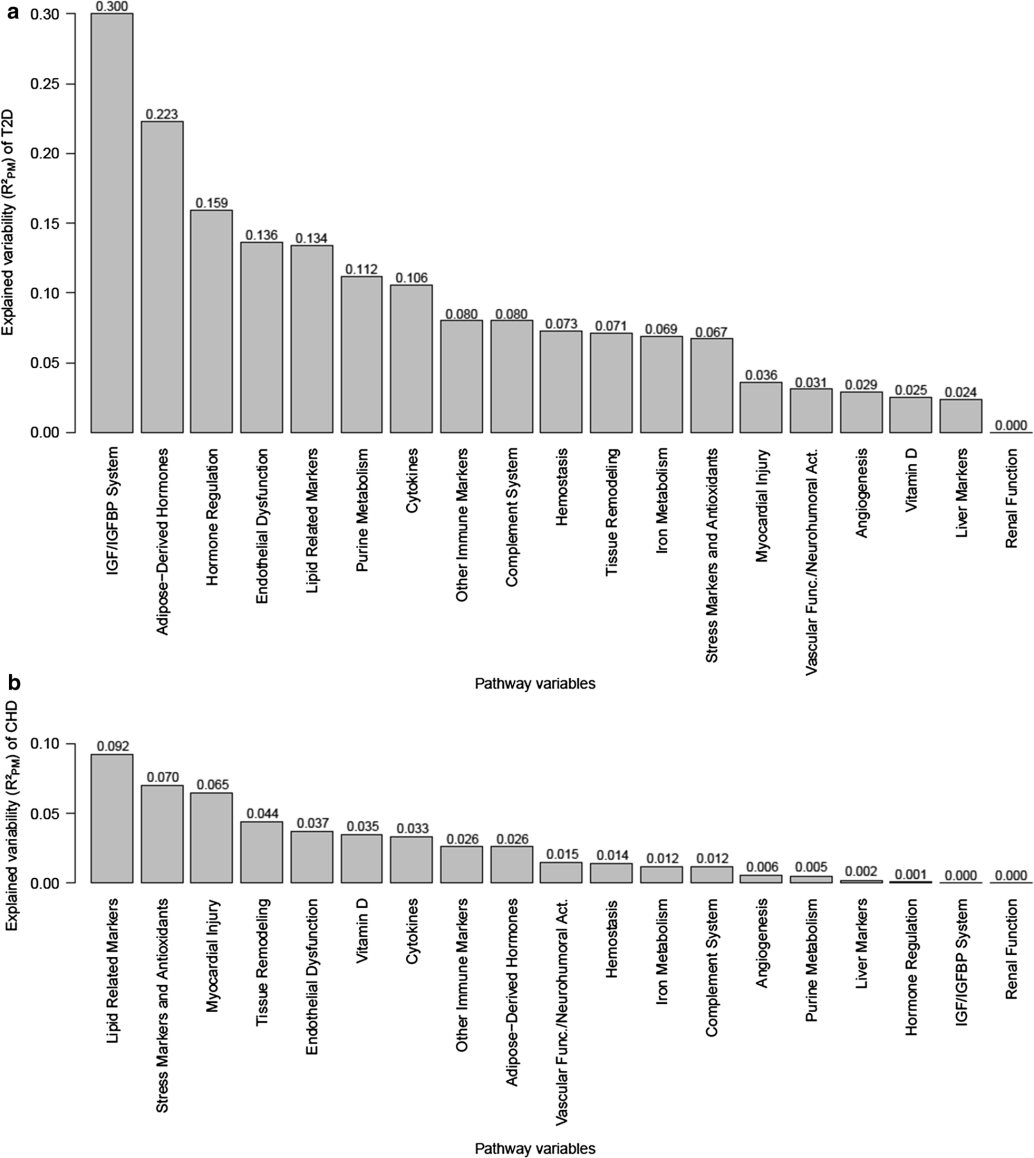
Explained variability (${\text{R}}^{ 2}_{\text{PM}}$) of **a** incident T2D and **b** incident CHD by single pathway variables when entered into empty models (univariate assessment)

All pathway variables together explained 49% of the T2D risk and 21% of the CHD risk. The magnitude explained by single pathway variables and their ranking regarding the importance for each disease varied greatly between T2D and CHD in the univariate assessment (Fig. 3a and 3b). Most of the T2D risk was explained by the IGF/IGFBP system pathway (represented by IGFBP-2, 30%), followed by the adipose-derived hormone pathway (represented by adiponectin, leptin, and resistin, 22%) and the hormone regulation pathway (represented by sex hormone binding globulin (SHBG), 16%). For CHD, most of the risk was explained by the lipid related pathway (represented by HDL-cholesterol, Lp(a), secretory phospholipase A2 group IIA (sPLA2-IIA), and total cholesterol, 9%), the stress and antioxidant pathway (represented by heat shock protein 70 (Hsp70), myeloperoxidase (MPO), and oxidized LDL (ox-LDL), 7%) as well as the myocardial injury pathway (represented by Troponin I, 7%). The endothelial dysfunction pathway (represented by soluble intercellular adhesion molecule-1 (sICAM-1) and soluble E-selectin (sE-selectin)) and lipid-related pathway were among the top five pathways for both diseases.

**Fig. 4:**
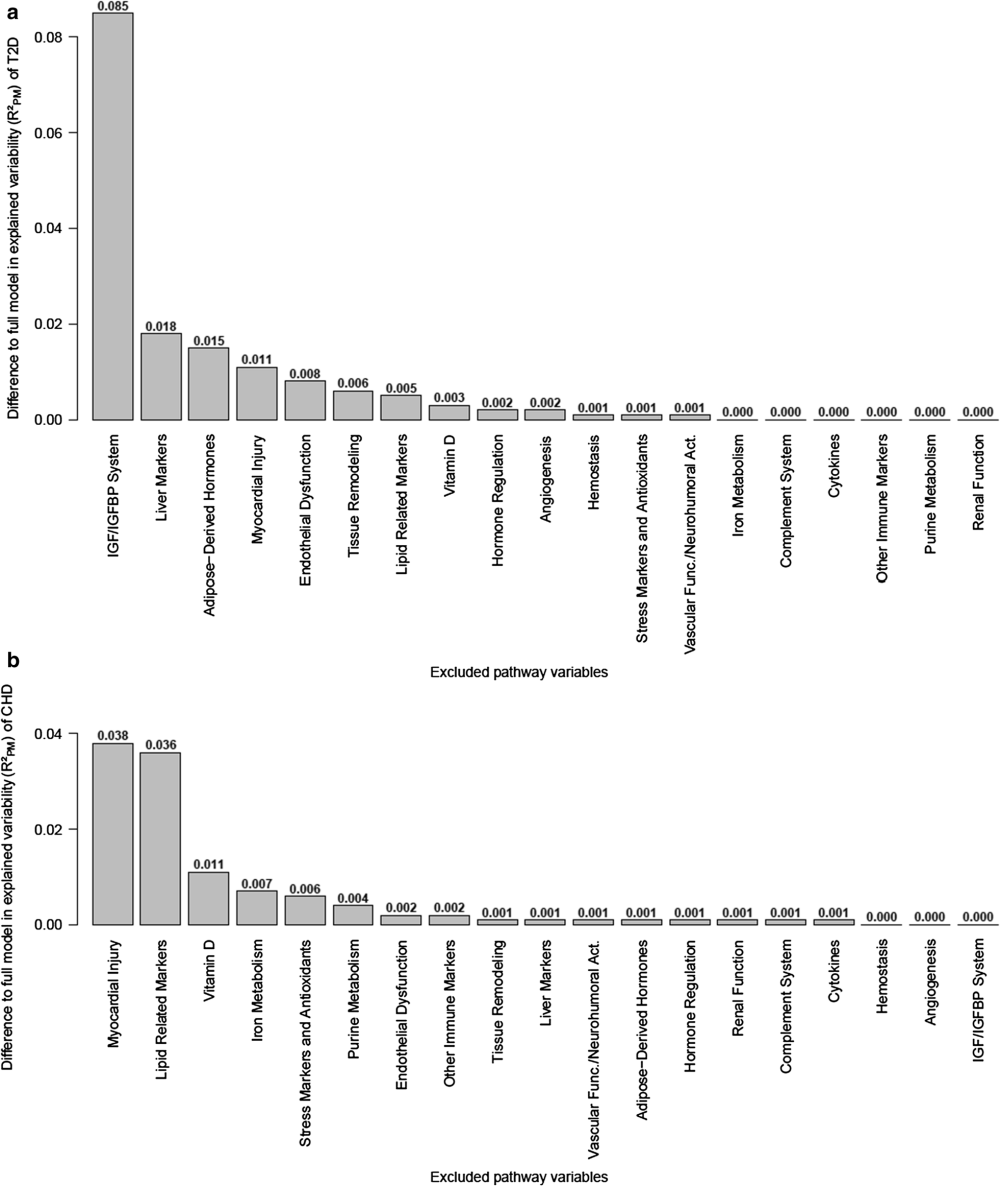
Absolute difference in explained variability (${\text{R}}^{ 2}_{\text{PM}}$) of **a** incident T2D and **b** incident CHD when single pathway variables were excluded from full models (assessment of independent contribution)

When evaluating the independent contributions of the pathways by excluding single pathway variables from the full model, the exclusion of the IGF/IGFBP pathway reduced the explained T2D risk by an absolute difference of 9%; all other pathway variables independently explained less than 2% (Fig. 4a). For CHD in contrast, two pathway variables, the myocardial injury and the lipid related pathways both independently explained 4% of the risk; all other pathway variables independently explained less than 2% (Fig. 4b).

## Discussion

In this large prospective study, we investigated biomarkers reflecting various pathophysiological processes for associations with both incident T2D as well as incident CHD. Our study is one of the first to examine to what extent biomarkers reflecting different etiology explain the development of T2D or CHD. Our study compared the results of the different pathways as well as of both diseases. We showed that the pathway variables collectively explained a substantially larger proportion of the T2D risk (49%) compared to CHD (21%). We also showed that the pathway variables that independently explained most of the disease risk differed between incident T2D and incident CHD. Moreover, the age, sex, and survey adjusted associations of some single biomarkers differed strongly for T2D and CHD, with Lp(a) and NT-proBNP even demonstrating opposite effect directions. However, the associations of many single biomarkers, such as several cytokines or the iron biomarker soluble transferrin receptor (sTfR), were quite similar for T2D and CHD.

### Importance of single pathways for T2D and CHD development in univariate assessment

The IGF/IGFBP system pathway was most strongly associated with T2D development in the univariate analyses, followed by the adipose-derived hormones and the hormone regulation pathways. For CHD, the lipid related, the stress and antioxidants, and the myocardial injury pathways individually were most important. Nonetheless, the associations of some single biomarkers were similar for T2D and CHD and the lipid related pathway (explained variability of T2D: 13%; CHD: 9%) and endothelial dysfunction pathway (T2D: 14%; CHD: 4%) both strongly explained the T2D as well as the CHD development when the pathways were individually investigated.

While the two biomarkers sE-selectin and sICAM-1 that represent the endothelial dysfunction pathway were positively associated with both T2D and CHD, more caution is required when interpreting the results from the lipid related pathway. Total cholesterol and sPLA2-IIA were both positively and HDL-cholesterol was inversely associated with incident T2D and CHD. Lp(a), however, was inversely associated with T2D and positively with CHD development. Accordingly, we inverted the age, sex, and survey adjusted Lp(a) residuals when we built the lipid related pathway variable for the T2D analysis, but not when we built this variable for the CHD analysis. This needs to be considered when interpreting the results. While it has been known for a long time that elevated Lp(a) increases the CHD risk, several prospective studies have shown more recently that the association with T2D is inverse [37–39]. The Lp(a) concentration is strongly genetically determined by the copy number variant kringle IV type 2 (KIV-2) and several single nucleotide polymorphisms in the *LPA* gene region [39]. Mendelian Randomization studies reported that genetic variants which lead to elevated Lp(a) levels were associated with higher CHD risk [39, 40] and lower T2D risk [39–42]. The results of these Mendelian Randomization studies strongly point at causality. However, the potentially causal mechanisms for both diseases, especially in relation to the observed opposite effect directions, are currently still obscure [37, 39]. Similar to the Lp(a) observation, the lipid marker low density lipoprotein (LDL) cholesterol also shows an inverse association with incident T2D and a positive association with incident CHD in observational [43, 44], clinical [9], and Mendelian Randomization studies [45]. The effects and side-effects of therapies aimed at lowering Lp(a) and LDL levels to reduce the risk of CHD are consequently a field of active research [39, 40, 46, 47]. According to the current state of knowledge, in individuals with elevated LDL levels being at increased risk of CVD, the CVD-preventive benefits of widespread LDL-lowering therapies clearly outweigh the increased T2D risk [39]. Unfortunately, LDL and triglyceride levels were not available in our study.

In our investigation, not only Lp(a), but also NT-proBNP, a marker of vascular function and neurohumoral activity, showed an inverse association with incident T2D and a positive association with incident CHD, which is consistent to the literature [48–50].

### Pathways independently associated with T2D or CHD

The analysis of single pathways revealed the strongest individual associations. However, these associations might partly be due to correlations to other pathway variables. We have therefore also investigated the independent contribution of each pathway by excluding one pathway variable at a time from a full model including all 19 pathway variables. None of the pathway variables independently and strongly explained both the development of T2D and CHD.

For T2D, only the IGF/IGFBP pathway independently explained the development of the disease strongly. This pathway, which was represented by IGFBP-2, had already shown the most pronounced association in the univariate assessment; increased IGFBP-2 serum levels conferred a decreased T2D risk. In recent years, evidence has accumulated that insulin-like growth factor 1 (IGF-1) and at least part of its binding proteins (IGFBPs) play an important role in glucose homeostasis [51]. The IGFBPs thereby act dependently and independently from IGF-1 [52]. Compared to IGF-1, IGFBP-1, and IGFBP-3, IGFBP-2 was most strongly associated with the development of T2D in women of the US Nurses’ Health Study [51]. The strong inverse association between IGFBP-2 and incident T2D that we also observed has been confirmed in three further studies [53–55]. In the Baltimore Longitudinal Study of Aging (BLSA) IGFBP-2 levels were positively correlated with insulin sensitivity at any time point during the study in men and women of middle to older age [56]. From a mechanistic point of view, IGFBP-2 may increase the insulin-stimulated glucose uptake by inducing glucose transporter 4 (GLUT-4) translocation from the cytoplasm to the plasma membrane [57]. Increased IGFBP-2 levels were also found to be associated with an increased risk of mortality in elderly persons [58, 59]—interestingly, though, in the BLSA study only after adjustment for insulin sensitivity [56].

The independent contribution of all other pathways was substantially weaker in the T2D analysis. Interestingly, the liver marker pathway, which was represented by fetuin-A (also known as alpha-2 Heremans Schmid glycoprotein), ranked on the second last place in the univariate assessment but ranked second best in the analysis of the independent contribution. The reason was that the estimated proportion of explained risk was only slightly attenuated when the other pathway variables were included in the model (from 2.4 to 1.8% explained risk), because the liver marker pathway was only weakly correlated with other important pathways. In our study, higher levels of fetuin-A were associated with an increased risk of T2D development, which a recent meta-analysis has also convincingly illustrated [15]. In this meta-analysis, the overall evidence and the five largest studies (including our own study) individually showed a positive association with T2D risk. The underlying mechanism likely involves insulin resistance caused by the impact of fetuin-A on diverse physiological processes, e.g. GLUT-4 translocation and protein kinase B (Akt) activation [60].

Concerning CHD, the effect of the stress and antioxidants pathway seen in the univariate assessment was strongly attenuated by the inclusion of the other pathway variables in the model (from 7.0 to 0.6%). In contrast, the myocardial injury and lipid related pathways were both independently strongly related to CHD development. The myocardial injury pathway was represented by ultra-sensitive troponin I, a marker of myocardial injury. Troponin I is used to diagnose acute MI in clinical practice and has also been shown to be predictive for incident CHD in individuals of the general population without prior CHD [17, 61].

### Impact

So far, only few studies have compared to what extent biomarkers that reflect different pathophysiological processes can explain the development of T2D or CHD relative to each other and even less, if any, have directly compared the relevance for both diseases. In their study, Montonen et al. used an effect decomposition method to compare how well the four most strongly associated biomarkers from four selected pathways explain the development of T2D [62]. Our approach extends this idea by mapping of substantially more pathophysiological processes and building of one average pathway variable to represent each process. We have applied the approach to our literature-based selected biomarker candidates, but future studies could utilize it similarly for the analysis of untargeted biomarker studies.

Our study has shown that some of the investigated biomarkers are associated with both T2D and CHD while some are only associated with one of the two diseases. In two instances, the effect direction even differed between the two studies. Moreover, the pathway variables that explained most of the disease risk differed between incident T2D and incident CHD. While there are a number of genetic studies that have examined comprehensively the common grounds of T2D and CHD [63, 64], we are not aware of other studies, which have investigated several biomarkers from diverse pathways simultaneously for both diseases. Consistent with our findings, the genetic studies overall show that only some variants contribute to both T2D and CVD development and that these genetic variants do not always share the same risk allele for T2D and CHD [63]. A recent large prospective study focused on a panel of nine inflammatory markers and compared their association with incident T2D and incident CVD. This study observed many similarities, but also important differences. For instance, the complement factor C3 was associated substantially more strongly with incident T2D than with incident CVD [65]. Our investigation included C3b, the larger of two elements formed when C3 is cleaved, and similarly observed a substantially stronger association with T2D compared to CHD. Altogether, the current evidence thus supports the existence of a common soil for both diseases, but also clearly shows that the common soil hypothesis has its limits. These limits deserve thorough investigation because particular caution is required if the treatment of one risk factor or disease might be harmful for another disease. Recent studies on LDL-lowering treatment have shown that this is not a far-fetched scenario, even for two strongly linked chronic diseases such as T2D and CHD [9]. In case opposing associations of pathophysiological mechanisms are detected, a careful evaluation of the harm and benefit of possible treatments is warranted. On the other hand, pathophysiological mechanisms, which are associated with several diseases in the same direction, are particular valuable candidates for further investigation, since treatments addressing these pathophysiological processes could have positive impacts on several outcomes.

We would like to point out that no conclusions on causality may be drawn from observational studies like our own. Thoroughly conducted Mendelian Randomization studies and eventually randomized clinical trials are necessary to prove the causality of the observed relationships. However, observational biomarker studies may contribute importantly to the development of pathophysiological hypotheses [63].

### Strengths and limitations

The main strength of our study is the prospective design, where all biomarkers were measured at baseline in non-diseased study participants. All cases developed during the long follow-up of 14 years. In order to minimize the risk of including T2D cases who were undiagnosed at baseline in the incident T2D analyses, all type 2 diabetic cases who were diagnosed during the first year after the baseline survey were excluded. A second strength is that we investigated many biomarkers from different metabolic pathways simultaneously. Noteworthy is also that we investigated T2D and CHD according to the same analysis approach in the same study population, which allows a direct comparison between the pathophysiological processes which play a role for these two frequent and highly related diseases. Another strength is the large sample size of our study, which includes more than 500 incident cases for each of the two investigated diseases. Last but not least, we developed a novel approach to investigate biomarker data from various pathways to get etiologic clues.

There are also some limitations that should be considered. This study has re-examined available biomarker data in their totality. This means that the biomarkers were not necessarily selected for the reason that they are representative for a specific pathway. It also means that the pathway variables were defined based on different numbers of biomarkers, which we have statistically taken into account by calculation of an average biomarker variable per pathway. Nevertheless, metabolic pathways with more biomarkers tend to be more representatively covered than pathways with only one or two biomarkers. For example, inclusion of additional biomarkers from the IGF/IGFBP system pathway such as IGF-1, IGF-2, IGFBP-1, and IGFBP-3 would have been helpful to better estimate the contribution of this system to the T2D and CVD risk. Moreover, the assignment to the pathophysiologic pathways was ambiguous for some biomarkers, and several biomarkers might also have fitted to other pathway groups. We are also aware that the investigation of pathway variables is a simplifying approach. Therefore, we analyzed the data not only on the level of the pathway but also on the level of the single biomarkers. Finally, the opposite inclusion direction of some biomarkers in the formation of the pathway variables for T2D and CHD limits the comparability between the two diseases for these pathways. In order to avoid erroneous conclusions, these differences are described and discussed in the manuscript.

## Conclusion

In our study, the biomarker-derived pathway variables collectively explained a substantially larger proportion of the risk of T2D compared to that of CHD. The ranking of the pathway variables differed between the two diseases: The IGF/IGFBP system pathway was by far most strongly associated with T2D (about 9% explained risk, independent of age, sex, and all other pathway variables). For CHD, the myocardial injury and lipid related pathways were most strongly associated and both independently explained about 4% of the CHD risk. Our analysis of the single biomarkers showed that the age, sex, and survey adjusted associations of many biomarkers were similar in strength for T2D and CHD, but there were also important differences. Lipoprotein (a) and NT-proBNP even demonstrated opposite effect directions. Our study thus adds to the evidence that there exists a common soil for both diseases, but also clearly shows that the common soil hypothesis has its limits. Our results help to better understand the pathophysiology of the two diseases, with the ultimate goal of pointing out targets for lifestyle intervention and drug development to ideally prevent both T2D and CHD development.

## Supplementary information

**Additional file 1: Text S1.** Comments on analytical approach. **Table S1.** Measurement methods, coefficients of variation (CV), missing values pre-imputation in the T2D and CHD case-cohort studies, and decisions on ln-transformations. **Table S2.** Baseline biomarker values of cases and non-cases in the T2D and CHD case-cohort studies. **Table S3.** Age, sex, and survey adjusted hazard ratios (HR) with 95% confidence intervals per standard deviation (SD) increase in biomarker concentration for incident T2D and incident CHD. **Figure S1.** Flowchart showing sample sizes and reasons for exclusions. **Figure S2.** Pearson’s correlations between the pathway variables, calculated in the subcohort of the T2D case-cohort study. **Figure S3.** Pearson’s correlations between the pathway variables, calculated in the subcohort of the CHD case-cohort study.

**Additional file 2: Figure S4 (a).** Pearson’s correlation coefficients r between the age, sex, and survey adjusted biomarker residuals, calculated in the subcohort of the T2D case-cohort study. **Figure S4 (b).** Pearson’s correlation coefficients r between the age, sex, and survey adjusted biomarker residuals, calculated in the subcohort of the CHD case-cohort study.

## Data Availability

The data are subject to national data protection laws and restrictions were imposed by the Ethics Committee of the Bavarian Chamber of Physicians to ensure data privacy of the study participants. Therefore, data cannot be made freely available in a public repository. However, data can be requested through an individual project agreement with KORA via the online portal KORA.passt (https://epi.helmholtz-muenchen.de/). Please contact the corresponding author Cornelia Huth in case of further questions.

## Abbreviations

CHD: Coronary heart disease
GLUT-4: Glucose transporter 4
HDL: High density lipoprotein
HR: Hazard ratio
IGF: Insulin-like growth factor
IGFBP: Insulin-like growth factor binding protein
KORA: Cooperative Health Research in the Region of Augsburg
LDL: Low density lipoprotein
Lp(a): Lipoprotein (a)
MI: Myocardial infarction
MONICA: Monitoring of Trends and Determinants in Cardiovascular Disease
NT-proBNP: N-terminal pro B-type natriuretic peptide
SD: Standard deviation
sE-selectin: Soluble E-selectin
sICAM-1: Soluble intercellular adhesion molecule-1
sPLA2-IIA: Secretory phospholipase A2 group IIA
T2D: Type 2 diabetes
t-PA: Tissue plasminogen activator
