# Supplementary material for: Biomarker-defined pathways for incident type 2 diabetes and coronary heart disease—a comparison in the MONICA/KORA study
Source: Cardiovasc Diabetol. 2020 Mar 12;19:32. doi: 10.1186/s12933-020-01003-w (PMC7066738; doi:10.1186/s12933-020-01003-w)
Supplement: Supplementary file 1 — Additional file 1: Text S1. Comments on analytical approach. Table S1. Measurement methods, coefficients of variation (CV), missing values pre-imputation in the T2D and CHD case-cohort studies, and decisions on ln-transformations. Table S2. Baseline biomarker values of cases and non-cases in the T2D and CHD case-cohort studies. Table S3. Age, sex, and survey adjusted hazard ratios (HR) with 95% confidence intervals per standard deviation (SD) increase in biomarker concentration for incident T2D and incident CHD. Figure S1. Flowchart showing sample sizes and reasons for exclusions. Figure S2. Pearson’s correlations between the pathway variables, calculated in the subcohort of the T2D case-cohort study. Figure S3. Pearson’s correlations between the pathway variables, calculated in the subcohort of the CHD case-cohort study. [file 12933_2020_1003_MOESM1_ESM.docx]

**Additional File 1** to manuscript ‘Biomarker-defined pathways for incident T2D and CHD – a comparison in the MONICA/KORA study’ (**Huth et al.,** *Cardiovascular Diabetology,* 2020)

**Text S1: Comments on analytical approach**

In our analysis of the pathway effects, we decided to calculate the proportion of explained disease variability instead of hazard ratio estimates. We considered this to be more appropriate due to the aggregated nature of the pathway variables. In order to calculate ‘average’ pathway variables, we inverted all biomarker variables for which increased serum levels were associated with a lower risk of incident disease. This decision was taken separately for the two outcomes. Because the published information was not sufficient for all biomarkers to take literature-based decisions, we used the effect estimates from our own study as information base. For the majority of the biomarkers, the estimates of the hazard ratios pointed into the same direction for incident T2D and incident CHD. However, the associations pointed in opposite directions for IGFBP-2 (IGF/IGFBP system), CD40 ligand (hemostasis pathway), ferritin (iron metabolism), Lp(a) (lipid related pathway), and NT-proBNP (vascular function and neurohumoral activity). We decided to include also the biomarkers into the pathway variables, which were non-significantly associated. Otherwise, pathways containing many biomarkers would have been privileged to remain in the analysis and to show strong effects purely by chance.

The basic variables age, sex, and survey explained much more CHD variability (46%) than T2D variability (17%). We therefore pre-standardized all biomarker variables, by eliminating the age, sex, and survey variability and used the resulting biomarker residual variables when building the pathway variables. The results of all analyses on these pathway variables are therefore independent of possible age, sex, and survey effects, similar to analyses which were adjusted for these basic variables. We did not pre-standardize or adjust for lifestyle risk factors or anthropometric variables such as physical activity, smoking or body adiposity, in order to avoid over-adjustment; the 19 pathway variables represent a large part of the pathophysiological effects, which the standard risk factors exert. Two examples are given in the following. Body adiposity is represented mainly by the adipose-derived hormone [1, 2] but also other pathway variables such as cytokines [3]. Smoking affects the biomarker levels of several investigated pathways, e.g. the hormone regulation, the lipid related, the vitamin D [4], and cytokines pathway [5].

**Additional references**

1. Nigro E, Scudiero O, Monaco ML, Palmieri A, Mazzarella G, Costagliola C, Bianco A, Daniele A. New insight into adiponectin role in obesity and obesity-related diseases. Biomed Res Int. 2014;2014:658913.

2. Mechanick JI, Zhao S, Garvey WT. Leptin, An Adipokine With Central Importance in the Global Obesity Problem. Glob Heart. 2018;13:113-27.

3. Fantuzzi G. Adipose tissue, adipokines, and inflammation. J Allergy Clin Immunol. 2005;115:911-9.

4. Kapoor D, Jones TH. Smoking and hormones in health and endocrine disorders. Eur J Endocrinol. 2005;152:491-9.

5. Calapai G, Caputi AP, Mannucci C, Russo GA, Gregg E, Puntoni R, Lowe F, McEwan M, Bassi A, Morandi S, Nunziata A. Cardiovascular biomarkers in groups of established smokers after a decade of smoking. Basic Clin Pharmacol Toxicol. 2009;104:322-8.

**Table S1: Measurement methods, coefficients of variation (CV), missing values pre-imputation in the T2D and CHD case-cohort studies, and decisions on ln-transformations. The biomarkers are sorted alphabetically by pathway group.**

| **Pathophysiological pathway** | **Serum biomarker** | **Abbreviated biomarker name** | **Measurement method** | **Intra-assay CV** | **Inter-assay CV** | **Missing values** | | **Ln-transf** |
| --- | --- | --- | --- | --- | --- | --- | --- | --- |
|  |  |  |  |  |  | **T2D** | **CHD** |  |
| Adipose-Derived Hormones | Adiponectin | Adiponectin | ELISA (Mercodia, Uppsala, Sweden) | <10% | <10% | 19**%** | 18**%** | yes |
|  | Leptin | Leptin | ELISA (Mercodia, Uppsala, Sweden) | <10% | <10% | 19**%** | 18**%** | yes |
|  | Resistin | Resistin | Ultrasensitive single-molecule counting assay (Erenna Immunoassay System, Singulex, Alameda, CA, USA) | 9% | 7%^1^ | 21**%** | 18**%** | yes |
| Angiogenesis | Vascular endothelial growth factor | VEGF | Ultrasensitive single-molecule counting assay (Erenna Immunoassay System, Singulex, Alameda, CA, USA) | 9% | 16% | 22**%** | 21**%** | yes |
|  | Vascular endothelial growth factor receptor 2 | VEGF-R2 | Ultrasensitive single-molecule counting assay (Erenna Immunoassay System, Singulex, Alameda, CA, USA) | 4% | 3%^1^ | 21**%** | 19**%** | no |
| Complement System | C3b | C3b | Ultrasensitive single-molecule counting assay (Erenna Immunoassay System, Singulex, Alameda, CA, USA) | 13% | 8%^1^ | 21**%** | 18**%** | no |
| Cytokines | Interleukin-1 receptor antagonist | IL-1RA | Ultrasensitive single-molecule counting assay (Erenna Immunoassay System, Singulex, Alameda, CA, USA) | 6% | 6%^1^ | 22**%** | 20**%** | yes |
|  | Interleukin-6 | IL-6 | Sandwich ELISA (CLB, Amsterdam, Netherlands) | <10% | <10% | 8**%** | 7**%** | yes |
|  | Interleukin-8 | IL-8 | Luminex Multiplex technology using a Luminex 100 analyzer (Luminex Corporation, Austin, TX) | <10% | 11% | 9**%** | 7**%** | yes |
|  | Interleukin-18 | IL-18 | Sandwich ELISA (CLB, Amsterdam, Netherlands) | <10% | 14% | 9**%** | 7**%** | yes |
|  | Interferon-gamma induced protein 10 | IP-10 | Luminex Multiplex technology using a Luminex 100 analyzer (Luminex Corporation, Austin, TX) | <10% | 35% | 9**%** | 7**%** | yes |
| **Pathophysiological pathway** | **Serum biomarker** | **Abbreviated biomarker name** | **Measurement method** | **Intra-assay CV** | **Inter-assay CV** | **Missing values**  **T2D CHD** | | **Ln-transf** |
|  | Monocyte chemotactic  protein-1 | MCP-1 | Luminex Multiplex technology using a Luminex 100 analyzer (Luminex Corporation, Austin, TX) | <10% | 20% | 9**%** | 7**%** | no |
|  | Macrophage migration inhibitory factor | MIF | Quantikine ELISA (R&D Systems, Wiesbaden, Germany) | 4% | 11% | 12**%** | 11**%** | yes |
|  | RANTES | RANTES | Quantikine ELISA (R&D Systems, Wiesbaden, Germany) | 5% | 6% | 12**%** | 11**%** | yes |
|  | Transforming growth factor beta | TGF-ß1 | Quantikine ELISA (R&D Systems, Wiesbaden, Germany) | 3% | 9% | 19**%** | 18**%** | no |
| Endothelial Dysfunction | Soluble E-Selectin | sE-selectin | Quantikine ELISA (R&D Systems, Wiesbaden, Germany) | 3% | 6% | 8**%** | 7**%** | yes |
|  | Soluble intercellular adhesion molecule-1 | sICAM-1 | ELISA (Diaclone, Besançon, France) | 2% | 5% | 8**%** | 7**%** | yes |
| Hemostasis | Alpha 2-anti-plasmin | Alpha 2AP | Ultrasensitive single-molecule counting assay (Erenna Immunoassay System, Singulex, Alameda, CA, USA) | 8% | 7%^1^ | 21**%** | 18**%** | no |
|  | CD40 ligand | CD40LG | Ultrasensitive single-molecule counting assay (Erenna Immunoassay System, Singulex, Alameda, CA, USA) | 14% | 10%^1^ | 21**%** | 18**%** | yes |
|  | Tissue plasminogen activator | t-PA | Ultrasensitive single-molecule counting assay (Erenna Immunoassay System, Singulex, Alameda, CA, USA) | 9% | 7%^1^ | 21**%** | 19**%** | yes |
| Hormone Regulation | Sex hormone binding globulin | SHBG | Ultrasensitive single-molecule counting assay (Erenna Immunoassay System, Singulex, Alameda, CA, USA) | 20% | 13%^1^ | 21**%** | 18**%** | yes |
| IGF/IGFBP System | Insulin-like growth factor binding protein 2 | IGFBP-2 | Ultrasensitive single-molecule counting assay (Erenna Immunoassay System, Singulex, Alameda, CA, USA) | 21% | 15%^1^ | 21**%** | 18**%** | yes |

| **Pathophysiological pathway** | **Serum biomarker** | **Abbreviated biomarker name** | **Measurement method** | **Intra-assay CV** | **Inter-assay CV** | **Missing values** | | **Ln-transf** |
| --- | --- | --- | --- | --- | --- | --- | --- | --- |
|  |  |  |  |  |  | **T2D** | **CHD** |  |
| Iron Metabolism | Ferritin | Ferritin | Ultrasensitive single-molecule counting assay (Erenna Immunoassay System, Singulex, Alameda, CA, USA) | 5% | 5%^1^ | 21**%** | 18**%** | yes |
|  | Soluble transferrin receptor | sTfR | Ultrasensitive single-molecule counting assay (Erenna Immunoassay System, Singulex, Alameda, CA, USA) | 5% | 5%^1^ | 21**%** | 18**%** | yes |
| Lipid Related Markers | High density lipoprotein cholesterol | HDL-cholesterol | Routine enzymatic method (CHOD-PAP; Boehringer Mannheim, Mannheim, Germany) | n.a.^2^ | n.a.^2^ | 0.04% | 0.04% | yes |
|  | Lipoprotein (a) | Lp(a) | Ultrasensitive single-molecule counting assay (Erenna Immunoassay System, Singulex, Alameda, CA, USA) | 6% | 9%^1^ | 21**%** | 18**%** | yes |
|  | Secretory phospho-lipase A2 group IIA | sPLA2-IIA | Ultrasensitive single-molecule counting assay (Erenna Immunoassay System, Singulex, Alameda, CA, USA) | 12% | 10%^1^ | 22**%** | 20**%** | yes |
|  | Total cholesterol | Total cholesterol | Routine enzymatic method (CHOD-PAP; Boehringer Mannheim, Mannheim, Germany) | n.a.^2^ | n.a.^2^ | 0.04% | 0.04% | yes |
| Liver Markers | Fetuin-A | Fetuin-A | Ultrasensitive single-molecule counting assay (Erenna Immunoassay System, Singulex, Alameda, CA, USA) | 10% | 6%^1^ | 21**%** | 18**%** | yes |
| Myocardial Injury | Troponin I | Troponin I | Ultrasensitive single-molecule counting assay (Erenna Immunoassay System, Singulex, Alameda, CA, USA) | 4% | 7% | 13**%** | 12**%** | yes |

| **Pathophysiological pathway** | **Serum biomarker** | **Abbreviated biomarker name** | **Measurement method** | **Intra-assay CV** | **Inter-assay CV** | **Missing values** | | **Ln-transf** |
| --- | --- | --- | --- | --- | --- | --- | --- | --- |
|  |  |  |  |  |  | **T2D** | **CHD** |  |
| Other Immune Markers | Chitinase 1 | Chitinase 1 | Ultrasensitive single-molecule counting assay (Erenna Immunoassay System, Singulex, Alameda, CA, USA) | 5% | 4%^1^ | 21**%** | 19**%** | yes |
|  | C-reactive protein | CRP | High-sensitivity immunoradiometric assay (IRMA in S1/S2: men aged 45–74 years; S3 all men and women) or a high-sensitivity latex-enhanced immunonephelometric assay (S1: men aged 35–44 and all women) on a BN II analyzer (Siemens, Eschborn, Germany). Both methods gave similar results when the same samples were analyzed [Ref Khuseyinova et al. Clin Chem, 2003]. | 4% IRMA;  3% nephel. | 12% IRMA;  5% nephel. | **2%** | 1**%** | yes |
|  | Osteopontin | OPN | Ultrasensitive single-molecule counting assay (Erenna Immunoassay System, Singulex, Alameda, CA, USA) | 6% | 8%^1^ | 21**%** | 19**%** | yes |
|  | Retinol binding protein 4 | RBP-4 | Ultrasensitive single-molecule counting assay (Erenna Immunoassay System, Singulex, Alameda, CA, USA) | 12% | 8%^1^ | 21**%** | 19**%** | yes |
| Purine Metabolism | Uric acid | Uric acid | Uricase method in S1 and S3 or enzymatic colorimetric reaction in S3 (Uric Acid PAP; Boehringer Mannheim). | n.a.^2^ | n.a.^2^ | 0.2**%** | 0.3**%** | no |
| Renal Function | Creatinine | Creatinine | Automated Jaffe method in S1 and S2 (Technicon, SMAC autoanalyzer) or an enzymatic method in S3 (creatinine PAP, Boehringer Mannheim). The enzymatic method was calibrated according to the Jaffe method. | n.a.^2^ | n.a.^2^ | 0.00% | 0.00% | no |

| **Pathophysiological pathway** | **Serum biomarker** | **Abbreviated biomarker name** | **Measurement method** | **Intra-assay CV** | **Inter-assay CV** | **Missing values** | | **Ln-transf** |
| --- | --- | --- | --- | --- | --- | --- | --- | --- |
|  |  |  |  |  |  | **T2D** | **CHD** |  |
| Stress Markers and Antioxidants | Heat shock protein 70 | Hsp70 | Ultrasensitive single-molecule counting assay (Erenna Immunoassay System, Singulex, Alameda, CA, USA) | 8% | 9%^1^ | 21**%** | 19**%** | yes |
|  | Myeloperoxidase | MPO | ELISA (Mercodia, Uppsala, Sweden) | <10% | <10% | 19**%** | 18**%** | yes |
|  | Oxidized LDL | ox-LDL | ELISA (Mercodia, Uppsala, Sweden) | <10% | <10% | 19**%** | 18**%** | no |
| Tissue Remodeling | Decorin | Decorin | Ultrasensitive single-molecule counting assay (Erenna Immunoassay System, Singulex, Alameda, CA, USA) | 6% | 4%^1^ | 21**%** | 19**%** | yes |
|  | Tumor necrosis factor receptor 6 | Fas receptor | Ultrasensitive, single-molecule counting assay (Erenna, Singulex, USA) | 20% | 16%^1^ | 21**%** | 19**%** | yes |
|  | Matrix metalloproteinase 8 | MMP8 | Ultrasensitive, single-molecule counting assay (Erenna, Singulex, USA) | 7% | 5%^1^ | 21**%** | 19**%** | yes |
|  | Matrix metalloproteinase 9 | MMP9 | Ultrasensitive, single-molecule counting assay (Erenna, Singulex, USA) | 6% | 5%^1^ | 21**%** | 18**%** | yes |
|  | Metallopeptidase inhibitor 4 | TIMP4 | Ultrasensitive, single-molecule counting assay (Erenna, Singulex, USA) | 9% | 7%^1^ | 21**%** | 19**%** | yes |
| Vascular Function and Neurohumoral Activity | Kallikrein | Kallikrein | Ultrasensitive, single-molecule counting assay (Erenna, Singulex, USA) | 4% | 3%^1^ | 21**%** | 19**%** | no |
|  | N-terminal pro B-type natriuretic peptide | NT-proBNP | Ultrasensitive, single-molecule counting assay (Erenna, Singulex) | 4% | 7%^1^ | 21**%** | 18**%** | yes |
| Vitamin D | 25-Hydroxy- cholecalciferol | 25(OH)D | Enzyme immunoassay OCTEIA (IDS, Frankfurt, Germany) | 3% | 6% | 31**%** | 30**%** | yes |

^1^The Singulex inter-assay coefficients of variation (CV) are reported after plate normalization, which was partly based on the control samples used for CV calculation.

^2^Intra- and inter-assay CVs are not presented because these biomarkers were measured from fresh serum continuously at the days of the baseline examinations.

Table S2: Baseline biomarker values of cases and non-cases in the T2D and CHD case-cohort studies. The biomarkers are sorted alphabetically by pathway group.

| **Pathophysiological pathway** | **Biomarker** [**unit**] | **Incident T2D** | | **Incident CHD** | |
| --- | --- | --- | --- | --- | --- |
|  |  | **Cases**  n = 689 | **Non-cases**  n = 1,850 | **Cases**  n = 568 | **Non-cases**  n = 2,004 |
| Adipose-Derived Hormones | Adiponectin [μg/mL] | 9.46 (7.24; 11.96) | 11.63 (8.89; 15.01) | 10.50 (8.03; 14.05) | 11.41 (8.75; 14.82) |
|  | Leptin [ng/mL] | 13.85 (6.99; 28.15) | 8.81 (4.66; 17.30) | 8.14 (4.84; 15.58) | 9.16 (4.84; 19.00) |
|  | Resistin [ng/mL] | 3.69 (2.75; 5.27) | 3.67 (2.72; 4.90) | 3.73 (2.79; 5.43) | 3.65 (2.71; 4.89) |
| Angiogenesis | VEGF [pg/mL] | 301.0 (183.2; 466.3) | 296.0 (175.3; 442.7) | 294.5 (179.5; 468.9) | 295.5 (174.5; 442.4) |
|  | VEGF-R2 [ng/mL] | 45.8 (40.5; 51.2) | 43.8 (39.0; 49.2) | 45.4 (39.9; 51.0) | 43.9 (39.0; 49.3) |
| Complement System | C3b [mg/mL] | 39.3 (32.8; 47.7) | 35.7 (29.3; 42.0) | 37.9 (31.4; 45.4) | 36.1 (29.7; 42.9) |
| Cytokines | IL-1RA [pg/mL] | 440.5 (343.1; 582.0) | 330.3 (269.9; 427.0) | 383.2 (307.0; 498.4) | 336.9 (272.4; 435.7) |
|  | IL-6 [pg/mL] | 3.21 (1.98; 4.82) | 2.19 (1.29; 3.52) | 3.20 (1.98; 4.95) | 2.21 (1.33; 3.56) |
|  | IL-8 [pg/mL] | 7.62 (5.51; 10.64) | 6.96 (5.01; 9.85) | 7.69 (5.57; 11.13) | 7.01 (5.04; 9.85) |
|  | IL-18 [pg/mL] | 199.3 (136.0; 282.3) | 172.9 (116.7; 246.9) | 182.0 (122.4; 267.5) | 174.9 (117.2; 248.3) |
|  | IP-10 [pg/mL] | 258.5 (160.7; 391.3) | 216.4 (140.1; 350.0) | 251.1 (151.6; 404.6) | 219.1 (142.1; 351.0) |
|  | MCP-1 [pg/mL] | 236.4 (144.5; 342.3) | 204.9 (123.9; 309.7) | 242.1 (150.0; 364.8) | 202.6 (125.7; 309.5) |
|  | MIF [ng/mL] | 18.5 (14.9; 23.7) | 17.8 (14.1; 22.5) | 19.1 (15.4; 24.0) | 17.8 (14.3; 22.5) |
|  | RANTES [ng/mL] | 23.9 (15.5; 36.5) | 23.4 (15.6; 36.1) | 24.2 (15.5; 35.0) | 23.4 (15.6; 36.8) |
|  | TGF-ß1 [ng/mL] | 35.2 (30.2; 40.6) | 34.8 (30.2; 40.0) | 34.2 (29.2; 39.3) | 34.8 (30.3; 40.2) |
| Endothelial Dysfunction | sE-selectin [ng/mL] | 64.9 (48.6; 85.0) | 51.0 (37.2; 65.9) | 58.6 (42.1; 78.2) | 51.8 (37.8; 67.4) |
|  | sICAM-1 [ng/mL] | 802.9 (642.0; 1003) | 713.9 (577.2; 878.0) | 813.2 (652.3; 1036) | 719.1 (581.8; 883.1) |

| **Pathophysiological pathway** | **Biomarker** [**unit**] | **Incident T2D** | | **Incident CHD** | |
| --- | --- | --- | --- | --- | --- |
|  |  | **Cases**  n = 689 | **Non-cases**  n = 1,850 | **Cases**  n = 568 | **Non-cases**  n = 2,004 |
| Hemostasis | Alpha 2AP [μg/mL] | 3.89 (2.52; 5.88) | 3.86 (2.52; 6.05) | 3.73 (2.42; 5.89) | 3.86 (2.53; 6.04) |
|  | CD40LG [ng/mL] | 16.2 (10.7; 24.7) | 17.4 (11.5; 26.1) | 15.6 (10.6; 24.4) | 17.5 (11.6; 26.0) |
|  | t-PA [ng/mL] | 8.17 (6.25; 10.28) | 5.47 (3.63; 7.74) | 7.94 (5.72; 10.01) | 5.59 (3.72; 7.94) |
| Hormone Regulation | SHBG [μg/mL] | 1.12 (0.73; 1.63) | 1.52 (1.03; 2.27) | 1.40 (0.91; 1.95) | 1.50 (1.00; 2.26) |
| IGF/IGFBP System | IGFBP-2 [ng/mL] | 39.8 (25.8; 62.8) | 62.9 (38.9; 97.0) | 65.5 (41.1; 110.0) | 59.7 (37.1; 94.6) |
| Iron Metabolism | Ferritin [ng/mL] | 356.2 (201.2; 660.1) | 240.0 (125.2; 449.5) | 349.1 (200.6; 617.0) | 253.3 (129.1; 482.7) |
|  | sTfR [μg/mL] | 1.09 (0.93; 1.26) | 1.03 (0.89; 1.19) | 1.09 (0.95; 1.27) | 1.03 (0.89; 1.20) |
| Lipid Related Markers | HDL-cholesterol [mg/dL] | 46.6 (39.1; 56.5) | 55.3 (45.8; 67.0) | 48.0 (39.9; 58.4) | 55.3 (45.6; 66.6) |
|  | Lp(a) [mg/dL]^1^ | 7.52 (4.08; 17.62) | 8.57 (4.76; 18.59) | 9.83 (4.96; 25.22) | 8.40 (4.71; 18.48) |
|  | sPLA2-IIA [ng/mL] | 9.05 (6.28; 13.00) | 8.25 (5.84; 11.48) | 8.86 (6.38; 12.77) | 8.45 (5.92; 11.75) |
|  | Total cholesterol [mg/dL] | 245.8 (214.8; 275.0) | 232.6 (206.7; 263.9) | 255.4 (225.8; 286.1) | 232.6 (206.4; 264.7) |
| Liver Markers | Fetuin-A [mg/mL] | 1.10 (0.96; 1.26) | 1.07 (0.94; 1.21) | 1.04 (0.93; 1.19) | 1.07 (0.94; 1.23) |
| Myocardial Injury | Troponin I [pg/mL] | 1.91 (1.30; 3.18) | 1.42 (0.93; 2.19) | 2.44 (1.63; 4.26) | 1.42 (0.94; 2.16) |
| Other Immune Markers | Chitinase 1 [ng/mL] | 55.0 (35.2; 79.2) | 48.2 (32.3; 73.8) | 64.6 (42.5; 92.4) | 47.3 (31.8; 71.0) |
|  | CRP [mg/L] | 2.57 (1.34; 4.92) | 1.33 (0.61; 2.88) | 2.51 (1.28; 5.57) | 1.35 (0.63; 3.04) |
|  | OPN [ng/mL] | 28.0 (16.5; 49.7) | 32.5 (19.5; 61.6) | 29.1 (17.2; 51.1) | 31.9 (19.2; 59.9) |
|  | RBP-4 [μg/mL] | 104.5 (84.0; 132.7) | 101.0 (81.9; 125.2) | 106.2 (82.3; 130.4) | 101.6 (82.5; 128.2) |
| Purine Metabolism | Uric acid [mg/dL] | 5.58 (4.64; 6.45) | 4.67 (3.76; 5.76) | 5.41 (4.48; 6.41) | 4.70 (3.80; 5.76) |
| Renal Function | Creatinine [mg/dL] | 0.90 (0.76; 1.07) | 0.87 (0.75; 1.02) | 0.95(0.79; 1.11) | 0.87 (0.75; 1.02) |

| **Pathophysiological pathway** | **Biomarker** [**unit**] | **Incident T2D** | | **Incident CHD** | |
| --- | --- | --- | --- | --- | --- |
|  |  | **Cases**  n = 689 | **Non-cases**  n = 1,850 | **Cases**  n = 568 | **Non-cases**  n = 2,004 |
| Stress Markers and Antioxidants | Hsp70 [ng/mL] | 4.20 (3.30; 5.54) | 3.83 (3.10; 5.00) | 4.24 (3.38; 5.47) | 3.85 (3.12; 5.04) |
|  | MPO [μg/mL] | 126.0 (98.1; 159.5) | 122.0 (95.2; 159.0) | 129.5 (99.9; 173.7) | 121.0 (94.1; 157.5) |
|  | ox-LDL [U/mL] | 103.0 (85.8; 122.0) | 88.8 (72.8; 106.0) | 106.0 (88.3; 123.0) | 89.1 (73.0; 107.0) |
| Tissue Remodeling | Decorin [ng/mL] | 13.9 (12.3; 15.7) | 12.9 (11.5; 14.5) | 13.8 (12.2; 15.5) | 13.0 (11.6; 14.6) |
|  | Fas receptor [ng/mL] | 0.53 (0.41; 0.70) | 0.48 (0.36; 0.64) | 0.54 (0.41; 0.75) | 0.48 (0.36; 0.65) |
|  | MMP8 [ng/mL] | 7.62 (5.40; 11.03) | 7.29 (4.95; 10.82) | 7.95 (5.55; 11.72) | 7.25 (4.93; 10.64) |
|  | MMP9 [ng/mL] | 399.0 (301.1; 526.1) | 381.6 (289.6; 508.2) | 405.2 (309.1; 529.6) | 380.7 (290.0; 508.5) |
|  | TIMP4 [ng/mL] | 4.82 (3.77; 6.03) | 4.72 (3.73; 5.89) | 5.05 (3.99; 6.63) | 4.72 (3.73; 5.92) |
| Vascular Function and Neurohumoral Activity | Kallikrein [μg/mL] | 37.0 (31.0; 43.7) | 36.3 (30.7; 41.8) | 36.7 (31.2; 42.9) | 36.5 (30.7; 42.2) |
|  | NT-proBNP [pg/mL]^1^ | 50.7 (26.7; 91.6) | 49.0 (28.7; 88.1) | 69.0 (35.0; 140.2) | 48.5 (27.9; 87.1) |
| Vitamin D | 25(OH)D [nmol/L] | 39.0 (28.7; 51.6) | 42.0 (31.3; 56.6) | 36.5 (27.4; 51.8) | 41.6 (31.1; 56.3) |

Biomarker values are presented as medians (25^th^ percentile; 75^th^ percentile)

^1^Lp(a) and NT-proBNP were calibrated using double measurements with another technique and Passing-Bablok regression, because the absolute concentrations generated by the ultrasensitive single-molecule counting Erenna Immunoassay System from Singulex (Alameda, CA, USA) showed an unusual value range. The calibration was performed on ln-transformed data; table S2 displays back-transformed values (anti-log) for descriptive purposes.

Details for Lp(a): Double measurements with the particle-enhanced turbidimetric immunoassay Biokit Quantia Lp(a)-Test from Abbott Diagnostics, USA, were available for *n* = 723 study participants. The Pearson correlation coefficient r between the ln-transformed Singulex and Abbott measurements was 0.92. Passing-Bablok regression yielded the calibration equation Lp(a)_calibrated = Lp(a)_Singulex x 0.7886 + 5.9241.

Details for NT-proBNP: Double measurements with the electrochemiluminescence immunoassay (ECLIA) Elecsys 2010, Modular Analytics E170, Cobas, from Roche, Mannheim, Germany, were available for *n* = 228 study participants. Pearson r between the ln-transformed Singulex and Roche measurements was 0.85. Passing-Bablok regression yielded the calibration equation NT-proBNP_calibrated = NT-proBNP_Singulex x 1.2665 – 2.9555.

Table S3: Age, sex, and survey adjusted hazard ratios (HR) with 95% confidence intervals (95% CI) per standard-deviation (SD) increase in biomarker concentration for incident T2D and incident CHD. The biomarkers are sorted alphabetically by pathway group. The last two columns specify whether the biomarkers were included positively or inversely in the creation of the pathway variables for the analyses of incident T2D and incident CHD.

| **Pathophysiological pathway** | **Biomarker** | **Incident T2D** | | **Incident CHD** | | **T2D** | **CHD** |
| --- | --- | --- | --- | --- | --- | --- | --- |
|  |  | **HR** [**95% CI**] | **p value** | **HR** [**95% CI**] | **p value** | **Association direction** | |
| Adipose-Derived Hormones | Adiponectin | 0.53 [0.47; 0.60] | <.0001 | 0.92 [0.80; 1.05] | 0.2286 | inverse | inverse |
|  | Leptin | 2.75 [2.31; 3.26] | <.0001 | 1.26 [1.08; 1.47] | 0.0039 | positive | positive |
|  | Resistin | 1.05 [0.95; 1.17] | 0.3480 | 1.12 [1.00; 1.26] | 0.0438 | positive | positive |
| Angiogenesis | VEGF | 1.08 [0.97; 1.21] | 0.1679 | 1.06 [0.93; 1.21] | 0.3547 | positive | positive |
|  | VEGF-R2 | 1.31 [1.18; 1.46] | <.0001 | 1.25 [1.11; 1.41] | 0.0002 | positive | positive |
| Complement System | C3b | 1.45 [1.30; 1.61] | <.0001 | 1.18 [1.05; 1.32] | 0.0039 | positive | positive |
| Cytokines | IL-1RA | 1.89 [1.70; 2.10] | <.0001 | 1.41 [1.26; 1.57] | <.0001 | positive | positive |
|  | IL-6 | 1.45 [1.30; 1.62] | <.0001 | 1.36 [1.20; 1.55] | <.0001 | positive | positive |
|  | IL-8 | 1.12 [1.01; 1.24] | 0.0274 | 1.12 [1.00; 1.27] | 0.0556 | positive | positive |
|  | IL-18 | 1.27 [1.14; 1.42] | <.0001 | 1.09 [0.97; 1.22] | 0.1491 | positive | positive |
|  | IP-10 | 1.15 [1.02; 1.29] | 0.0184 | 1.05 [0.92; 1.20] | 0.4642 | positive | positive |
|  | MCP-1 | 1.14 [1.04; 1.26] | 0.0073 | 1.12 [1.01; 1.24] | 0.0361 | positive | positive |
|  | MIF | 1.11 [1.00; 1.23] | 0.0483 | 1.17 [1.04; 1.32] | 0.0070 | positive | positive |
|  | RANTES | 1.09 [0.98; 1.22] | 0.1078 | 1.06 [0.94; 1.20] | 0.3608 | positive | positive |
|  | TGF-ß1 | 1.10 [0.98; 1.22] | 0.1005 | 1.01 [0.90; 1.13] | 0.9164 | positive | positive |
| Endothelial Dysfunction | sE-selectin | 1.83 [1.62; 2.05] | <.0001 | 1.27 [1.12; 1.44] | 0.0002 | positive | positive |
|  | sICAM-1 | 1.37 [1.23; 1.52] | <.0001 | 1.33 [1.18; 1.51] | <.0001 | positive | positive |

| **Pathophysiological pathway** | **Biomarker** | **Incident T2D** | | **Incident CHD** | | **T2D** | **CHD** |
| --- | --- | --- | --- | --- | --- | --- | --- |
|  |  | **HR** [**95% CI**] | **p value** | **HR** [**95% CI**] | **p value** | **Association direction** | |
| Hemostasis | Alpha 2AP | 1.02 [0.91; 1.15] | 0.6732 | 1.03 [0.91; 1.15] | 0.6526 | positive | positive |
|  | CD40LG | 1.00 [0.90; 1.11] | 0.9619 | 1.04 [0.93; 1.16] | 0.5347 | inverse | positive |
|  | t-PA | 2.48 [2.16; 2.86] | <.0001 | 1.55 [1.35; 1.79] | <.0001 | positive | positive |
| Hormone Regulation | SHBG | 0.54 [0.47; 0.61] | <.0001 | 0.90 [0.79; 1.04] | 0.1487 | inverse | inverse |
| IFG/IGFBP System | IGFBP-2 | 0.42 [0.37; 0.49] | <.0001 | 1.00 [0.89; 1.14] | 0.9600 | inverse | positive |
| Iron Metabolism | Ferritin | 1.45 [1.27; 1.66] | <.0001 | 0.99 [0.86; 1.14] | 0.8688 | positive | inverse |
|  | sTfR | 1.15 [1.04; 1.27] | 0.0076 | 1.18 [1.05; 1.33] | 0.0055 | positive | positive |
| Lipid Related Markers | HDL-cholesterol | 0.58 [0.51; 0.64] | <.0001 | 0.72 [0.64; 0.81] | <.0001 | inverse | inverse |
|  | Lp(a) | 0.85 [0.76; 0.96] | 0.0068 | 1.16 [1.03; 1.30] | 0.0138 | inverse | positive |
|  | sPLA2-IIA | 1.17 [1.07; 1.28] | 0.0010 | 1.21 [1.08; 1.36] | 0.0009 | positive | positive |
|  | Total cholesterol | 1.14 [1.03; 1.26] | 0.0136 | 1.51 [1.34; 1.70] | <.0001 | positive | positive |
| Liver Markers | Fetuin-A | 1.22 [1.10; 1.34] | 0.0001 | 1.04 [0.93; 1.16] | 0.5458 | positive | positive |
| Myocardial Injury | Troponin I | 1.37 [1.23; 1.52] | <.0001 | 1.59 [1.39; 1.81] | <.0001 | positive | positive |
| Other Immune Markers | Chitinase 1 | 0.93 [0.84; 1.03] | 0.1650 | 1.06 [0.93; 1.21] | 0.3466 | inverse | positive |
|  | CRP | 1.59 [1.46; 1.74] | <.0001 | 1.47 [1.32; 1.63] | <.0001 | positive | positive |
|  | OPN | 0.85 [0.76; 0.94] | 0.0024 | 1.03 [0.92; 1.15] | 0.6301 | inverse | positive |
|  | RBP-4 | 1.10 [0.98; 1.23] | 0.1203 | 1.02 [0.90; 1.15] | 0.7611 | positive | positive |
| Purine Metabolism | Uric acid | 1.68 [1.50; 1.87] | <.0001 | 1.17 [1.04; 1.32] | 0.0099 | positive | positive |
| Renal Function | Creatinine | 0.99 [0.90; 1.10] | 0.8905 | 1.05 [0.95; 1.16] | 0.3727 | inverse | positive |

| **Pathophysiological pathway** | **Biomarker** | **Incident T2D** | | **Incident CHD** | | **T2D** | **CHD** |
| --- | --- | --- | --- | --- | --- | --- | --- |
|  |  | **HR** [**95% CI**] | **p value** | **HR** [**95% CI**] | **p value** | **Association direction** | |
| Stress Markers and Antioxidants | Hsp70 | 1.21 [1.09; 1.33] | 0.0002 | 1.18 [1.06; 1.31] | 0.0034 | positive | positive |
|  | MPO | 1.12 [1.02; 1.22] | 0.0217 | 1.27 [1.14; 1.41] | <.0001 | positive | positive |
|  | ox-LDL | 1.50 [1.34; 1.68] | <.0001 | 1.47 [1.31; 1.66] | <.0001 | positive | positive |
| Tissue Remodeling | Decorin | 1.56 [1.39; 1.74] | <.0001 | 1.24 [1.11; 1.39] | 0.0001 | positive | positive |
|  | Fas receptor | 1.10 [1.00; 1.21] | 0.0401 | 1.14 [1.01; 1.28] | 0.0291 | positive | positive |
|  | MMP8 | 1.17 [1.07; 1.29] | 0.0011 | 1.33 [1.19; 1.50] | <.0001 | positive | positive |
|  | MMP9 | 1.18 [1.07; 1.31] | 0.0014 | 1.27 [1.13; 1.43] | <.0001 | positive | positive |
|  | TIMP4 | 0.92 [0.82; 1.02] | 0.1154 | 1.04 [0.92; 1.18] | 0.4993 | inverse | positive |
| Vascular Function and Neurohumoral Activity | Kallikrein | 1.16 [1.04; 1.29] | 0.0078 | 1.12 [0.98; 1.27] | 0.1005 | positive | positive |
|  | NT-proBNP | 0.85 [0.75; 0.97] | 0.0134 | 1.20 [1.06; 1.37] | 0.0050 | inverse | positive |
| Vitamin D | 25(OH)D | 0.80 [0.71; 0.89] | <.0001 | 0.73 [0.64; 0.83] | <.0001 | inverse | inverse |


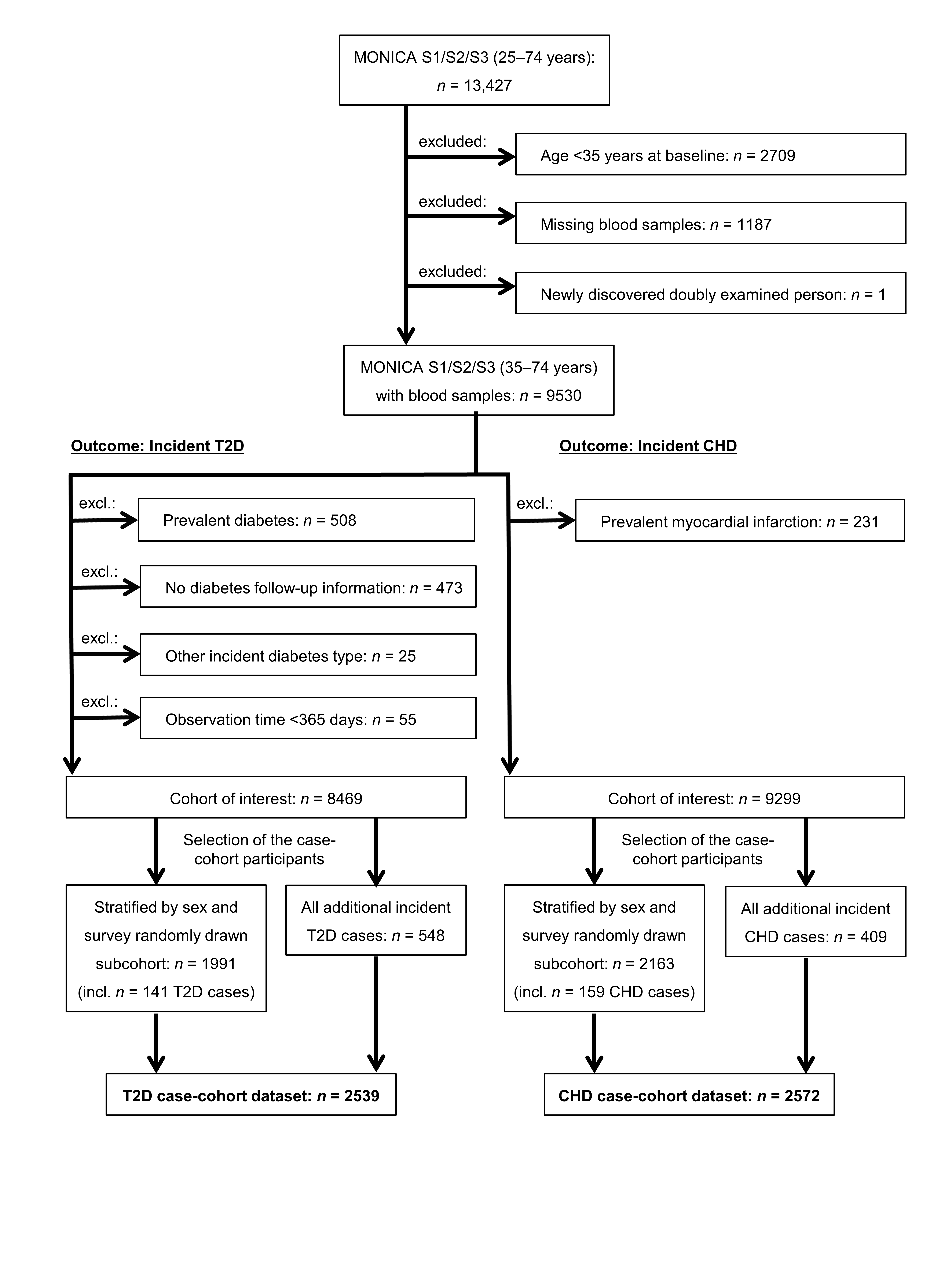


Figure S1: Flowchart showing sample sizes and reasons for exclusions


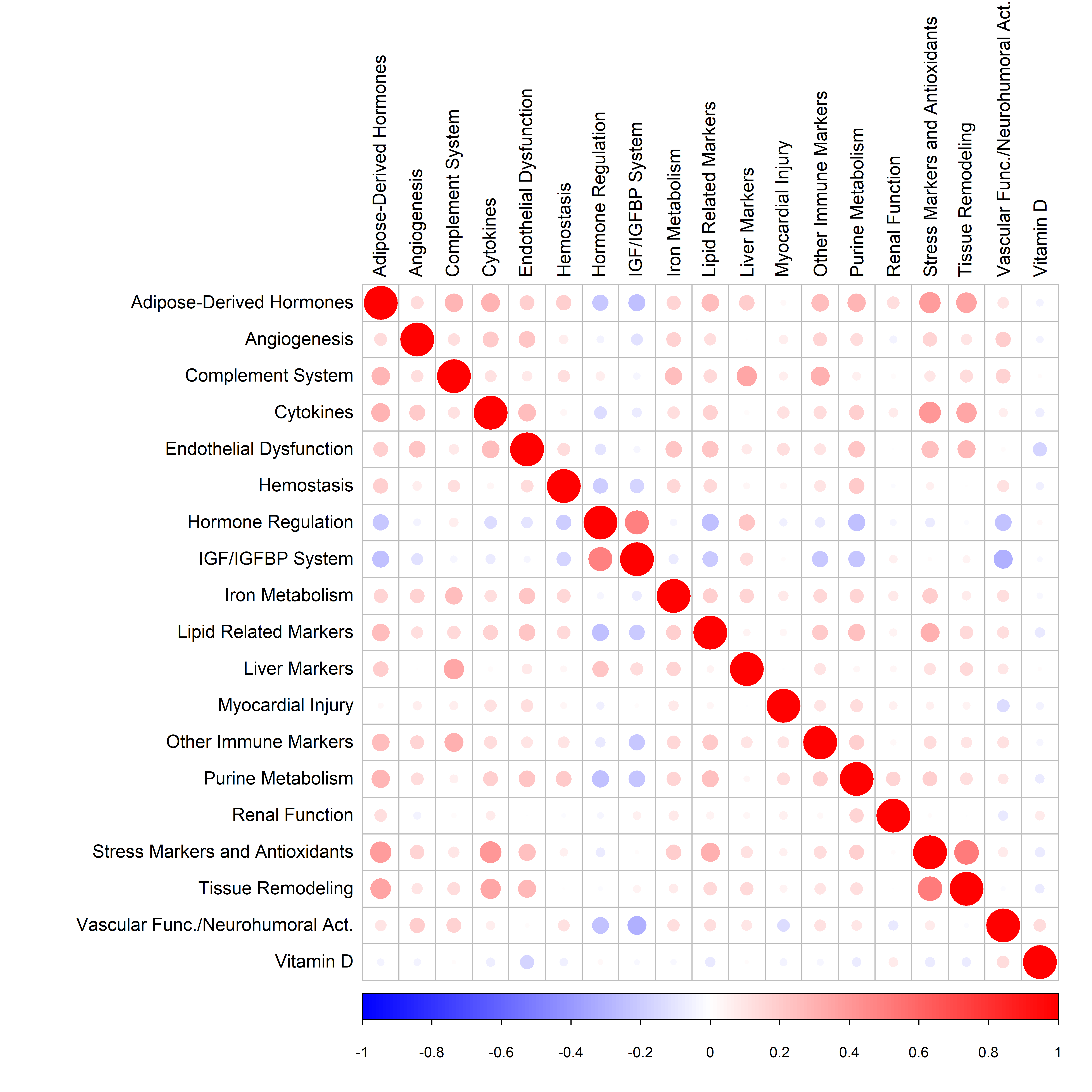


Figure S2: Pearson’s correlations between the pathway variables, calculated in the subcohort of the T2D case-cohort study


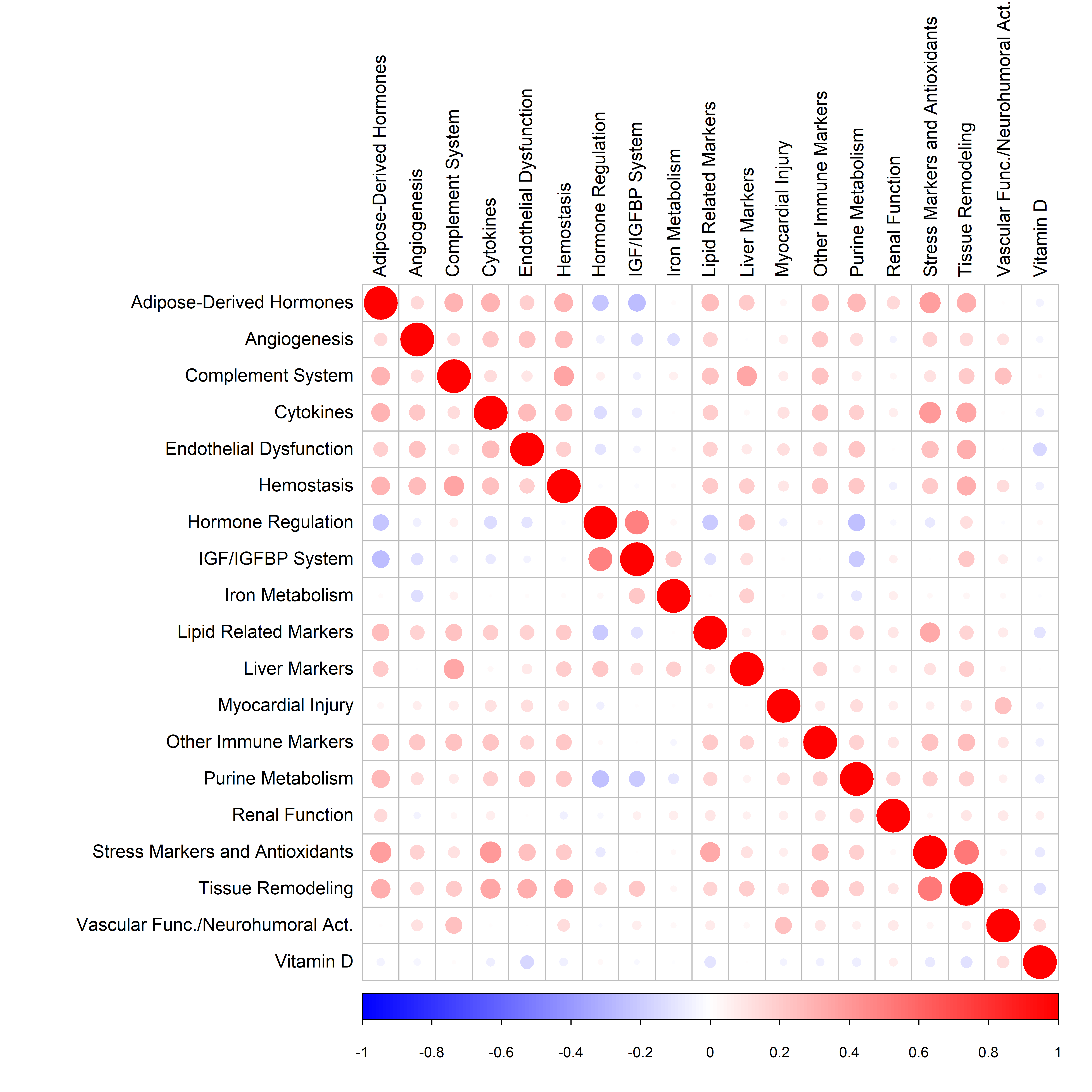


Figure S3: Pearson’s correlations between the pathway variables, calculated in the subcohort of the CHD case-cohort study
